# Racial/Ethnic Disparities in Mortality Across the Veterans Health Administration

**DOI:** 10.1089/heq.2018.0086

**Published:** 2019-04-08

**Authors:** Michelle S. Wong, Katherine J. Hoggatt, W. Neil Steers, Susan M. Frayne, Alexis K. Huynh, Elizabeth M. Yano, Fay S. Saechao, Boback Ziaeian, Donna L. Washington

**Affiliations:** ^1^ VA HSR&D Center for the Study of Healthcare Innovation, Implementation & Policy (CSHIIP), VA Greater Los Angeles Healthcare System, Los Angeles, California.; ^2^ Department of Epidemiology, UCLA Fielding School of Public Health, Los Angeles, California.; ^3^ Division of General Internal Medicine and Health Services Research, Department of Medicine, UCLA Geffen School of Medicine, Los Angeles, California.; ^4^ VA HSR&D Center for Innovation to Implementation (Ci2i), VA Palo Alto Health Care System, Menlo Park, California.; ^5^ Division of Primary Care and Population Health, Stanford University School of Medicine, Stanford, California.; ^6^ Department of Health Policy and Management, UCLA Fielding School of Public Health, Los Angeles, California.; ^7^ Division of Cardiology, Department of Medicine, UCLA Geffen School of Medicine, Los Angeles, California.

**Keywords:** health disparities, mortality, racial/ethnic disparities, Veterans

## Abstract

**Purpose:** Equal-access health care systems such as the Veterans Health Administration (VHA) reduce financial and nonfinancial barriers to care. It is unknown if such systems mitigate racial/ethnic mortality disparities, such as those well documented in the broader U.S. population. We examined racial/ethnic mortality disparities among VHA health care users, and compared racial/ethnic disparities in VHA and U.S. general populations.

**Methods:** Linking VHA records for an October 2008 to September 2009 national VHA user cohort, and National Death Index records, we assessed all-cause, cancer, and cardiovascular-related mortality through December 2011. We calculated age-, sex-, and comorbidity-adjusted mortality hazard ratios. We computed sex-stratified, age-standardized mortality risk ratios for VHA and U.S. populations, then compared racial/ethnic disparities between the populations.

**Results:** Among VHA users, American Indian/Alaskan Natives (AI/ANs) had higher adjusted all-cause mortality, whereas non-Hispanic Blacks had higher cause-specific mortality versus non-Hispanic Whites. Asians, Hispanics, and Native Hawaiian/Other Pacific Islanders had similar, or lower all-cause and cause-specific mortality versus non-Hispanic Whites. Mortality disparities were evident in non-Hispanic-Black men compared with non-Hispanic White men in both VHA and U.S. populations for all-cause, cardiovascular, and cancer (cause-specific) mortality, but disparities were smaller in VHA. VHA non-Hispanic Black women did not experience the all-cause and cause-specific mortality disparity present for U.S. non-Hispanic Black women. Disparities in all-cause and cancer mortality existed in VHA but not in U.S. population AI/AN men.

**Conclusion:** Patterns in racial/ethnic disparities differed between VHA and U.S. populations, with fewer disparities within VHAs equal-access system. Equal-access health care may partially address racial/ethnic mortality disparities, but other nonhealth care factors should also be explored.

## Introduction

Health care access is an important—though not sole—determinant of health outcomes.^1^ Racial/ethnic minorities face numerous barriers to accessing health services, including living in communities with fewer primary health care providers^2^ and being less likely to have a usual source of care.^3^ Equal-access health care systems—which strive to eliminate financial barriers to health care—may potentially mitigate well-documented racial/ethnic mortality disparities in the United States.^4^

The Veterans Health Administration (VHA) is an equal-access system that provides care to eligible Veterans without the requirement to pay insurance premiums. Because Veterans often face other nonfinancial barriers to accessing care (e.g., transportation, no sick time from work), VHA has other characteristics aimed to further increase access, such as transportation services to VHA facilities, extended evening clinic hours, and comprehensive physical and mental health services. These characteristics may also help mitigate racial/ethnic health disparities.

VHA, the largest equal-access systems in the United States, is a suitable model to examine whether equal-access systems and efforts to address nonfinancial barriers to care can reduce racial/ethnic mortality disparities relative to those in the general population. While non-Hispanic Blacks in the U.S. general population experience higher mortality than non-Hispanic Whites,^5^ studies in subgroups of Veterans suggest that these disparities are mitigated among VHA users.^6,7^

The persistence of racial/ethnic mortality disparities in the United States, though, warrants continued research within equal-access systems, such as VHA, to understand the role of these systems—and health care access more broadly—to address this issue. There are gaps in the research on mortality disparities even among extant VHA-based studies, which have been limited to specific geographical regions or subpopulations (e.g., patients with type 2 diabetes). To our knowledge, no study has examined racial/ethnic disparities for all-cause and leading cause-specific mortality, that is, cancer and cardiovascular disease,^8^ which differentially affect racial/ethnic groups,^4^ in the national population of all VHA users. In addition, while most VHA studies have focused on non-Hispanic Black–non-Hispanic White disparities, few studies have considered mortality disparities in other racial/ethnic groups.^9^

This analysis had two main aims: first, we characterized racial/ethnic disparities in all-cause, cancer, and cardiovascular (cause-specific) mortality within VHA for American Indian/Alaskan Natives (AI/ANs), Asians, non-Hispanic Blacks, Hispanics, and Native Hawaiians/Other Pacific Islanders (NH/OPIs), compared with non-Hispanic Whites. We anticipated that mortality rates within VHA for all-cause, cancer, and cardiovascular mortality would be similar for all racial/ethnic minority groups compared with non-Hispanic Whites, given existing evidence of attenuated non-Hispanic Black–non-Hispanic White mortality disparities within VHA for selected subgroups. Second, we compared racial/ethnic mortality disparities within the U.S. general population with those among Veterans receiving care from VHA. Racial/ethnic disparities are established in the U.S. general population, particularly between non-Hispanic Blacks and non-Hispanic Whites,^8^ but we anticipated that disparities would be attenuated within the equal-access VHA, given its efforts to improve access to VHA users.

## Methods

### Data and sample

Our analytical sample was a national cohort of all Veterans, age ≥18, who had at least one VHA ambulatory care visit from October 2008 to September 2009 (fiscal year [FY]2009). We used data from VHAs electronic medical records linked to the Centers for Disease Control and Prevention (CDC) National Death Index (NDI). Patient records were linked based on exact matches of social security numbers and date of birth. We excluded individuals with duplicate NDI records, invalid dates of index ambulatory care visits (*n*=4778), or missing covariates (*n*=66). Mortality was ascertained from October 2008 to December 2011. Details about the analytical sample are available in the Supplementary Data.

We calculated U.S. national comparator age-adjusted sex-stratified overall and cause-specific mortality rates stratified by race/ethnicity using the National Center for Health Statistics (NCHS)'s detailed Mortality files, and age-adjusted using U.S. Census annual population estimates.^10^ We excluded Asians and NH/OPIs in our comparison of mortality disparities between VHA and U.S. general population because the CDC's mortality files do not disaggregate these two groups.

### Measures

Dependent variables were all-cause mortality, cancer mortality, and cardiovascular mortality measures. Aim 1 examined time to all-cause, cancer, and cardiovascular-related mortality, calculated as the difference in years between date of death and qualifying date of initial FY2009 ambulatory care visit. Aim 2 assessed mortality proportions (mortality count/population). We identified cancer mortality from the NDI through malignant neoplasm ICD-10 codes (C00–C97) and cardiovascular-related mortality through diseases of the heart ICD-10 codes (I00–I09, I11, I13, I20–I51).

Our main predictor of interest was a categorical indicator of patient race/ethnicity. Patient race/ethnicity in the VHA population was ascertained from multiple VHA and U.S. Department of Defense databases using a previously described algorithm.^11^ All individuals with Hispanic ethnicity were classified as Hispanic; all others were classified by race. We considered the following racial/ethnic groups: AI/AN, Asian, non-Hispanic Black, Hispanic, NH/OPI, and non-Hispanic White (reference group). In the U.S. population, race/ethnicity for mortality counts came from death records, and for population counts, from self-identified race/ethnicity in the U.S. Census.

To examine disparities within the VHA, we included covariates for age (categorical: 18–29, 30–39, 40–49, 50–59, 60–69, 70–79, and 80+), sex, medical comorbidity, and mental health comorbidity. We constructed a medical comorbidity index, adapted from the Seattle Index of Comorbidity,^12^ based on a weighted count of smoking status and seven chronic medical conditions associated with increased mortality (prior myocardial infarction, cancer, lung disease, congestive heart failure, diabetes, pneumonia, and stroke). Mental health comorbidity was a hierarchical four-category variable: serious mental illness (SMI, defined as a diagnosis of schizophrenia, schizoaffective or bipolar disorder, or other psychoses), depression without SMI, other mental health diagnosis without SMI or depression, and no mental health diagnoses. We identified medical and mental health diagnoses using ICD-9-CM outpatient and inpatient diagnosis codes from FY2009.

### Statistical analysis

We calculated descriptive statistics of race/ethnicity-stratified means and proportions of the VHA population's demographic and health characteristics. We calculated race/ethnicity-stratified crude mortality rates, and age- and sex-standardized (to the age and sex distribution of non-Hispanic White FY2009 Veterans VHA users) mortality proportions for all-cause, cancer, and cardiovascular-related mortality, as the number of deaths per 100,000 population.

#### Disparities within the VHA

We examined racial/ethnic disparities in mortality rates within VHA using Cox regression models to calculate hazard ratios of mortality that compared each racial/ethnic group with non-Hispanic Whites. Deaths were classified as either observed on the date of death or administratively censored at the end of the mortality ascertainment period. For each outcome, we ran two models: model 1 included age and sex; model 2 included age, sex, and medical and mental health comorbidities. We used alpha=0.05 as the threshold for statistical significance.

#### Comparison with the U.S. general population

Because comparisons of racial/ethnic disparities in mortality between VHA and the U.S. general population are confounded by between-group differences in the age and sex compositions, we computed sex-stratified, age-standardized mortality “risk” ratios (RRs) for minority racial/ethnic groups compared with the non-Hispanic White group, using the previously described seven age categories. Age was determined as of FY2009 for VHA users and from 2010 census for the U.S. general population. To compare the age-standardized RRs between VHA and the U.S. general population, we used one set of age standardization weights for VHA and U.S. men (based on the age distribution of non-Hispanic White, male VHA patients) and one set of age standardization weights for VHA and U.S. women (based on the age distribution of non-Hispanic White, female VHA patients). We limited this analysis to racial/ethnic groups for whom mortality and population counts were available in the U.S. data (non-Hispanic Whites, AI/ANs, non-Hispanic Blacks, and Hispanics). We computed both point and 95% interval estimates for the standardized RRs for the VHA and U.S. population. We considered nonoverlapping confidence intervals (CIs) for the RRs, for VHA versus the U.S. population, as indicating a statistical difference in the RRs.^13^ All statistical analyses were conducted in Stata 15 (Stata-Corp, College Station, TX).

This program evaluation work received a Determination of Non-Research from VA Greater Los Angeles Healthcare System Institutional Review Board Research and Development Committee.

## Results

The cohort of 5,030,656 Veterans were at risk and observed for 14,442,378 person-years, during which 516,540 deaths were observed. Table 1 presents demographic and health characteristics of the cohort. Approximately three-quarters of Veterans were non-Hispanic White,14.0% non-Hispanic Black, 4.8% Hispanic, 0.7% Asian, 0.6% NH/OPI, and 0.5% AI/AN. Non-Hispanic Whites were oldest (mean 65.1 years, SD: 15.2). Overall, 5.7% of Veterans were female. Asians had the lowest mean medical morbidity score (1.3, SD: 1.8). Non-Hispanic Blacks had the highest proportion of individuals carrying a diagnosis of SMI (7.5%), whereas Asians had the highest proportion of individuals without a mental health diagnosis (75.3%).

**Table 1.** Veteran Health Administration User Sample Characteristics

	Non-Hispanic White 78.2% (*n*=3,766,450)	AI/AN 0.5% (*n*=23,330)	Asian 0.7% (*n*=33,890)	Non-Hispanic Black 14.0% (*n*=718,720)	Hispanic 4.8% (*n*=242,349)	NH/OPI 0.6% (*n*=29,277)
Age, mean (SD)	65.1 (15.2)	57.6 (15.1)	56.3 (19.0)	56.5 (14.0)	57.0 (17.4)	60.3 (15.9)
Female, %	4.4	8.3	7.4	9.9	6.0	7.6
Medical comorbidity,^a^ mean (SD)	1.9 (2.2)	1.9 (2.3)	1.3 (1.8)	1.9 (2.2)	1.6 (2.0)	1.9 (2.2)
Mental health comorbidity, %						
SMI^b^	4.26	5.5	4.3	7.5	6.5	5.8
Depression without SMI	18.3	23.4	13.4	18.3	21.3	20.4
Other mental health disorders without SMI or depression	6.7	13.5	7.1	11.8	9.9	10.4
No mental health diagnosis	70.7	57.6	75.3	62.5	62.6	63.5
Crude mortality proportion per 100,000^c^						
All cause	11,486.9	8444.1	5340.8	7309.7	6923.5	9109.5
Cancer^d^	2934.5	2216.0	1416.3	2119.3	1625.8	2466.1
Cardiovascular related^e^	3073.4	1813.1	1292.4	1905.2	1584.9	2452.4
Age- and sex-standardized mortality proportion per 100,000^c,f^						
All cause	11,486.9	12,952.9	7386.5	12,592.7	10,116.8	11,619.3
Cancer^d^	2934.5	3307.7	1981.6	3536.7	2315.1	3084.9
Cardiovascular related^e^	3073.4	3008.0	1802.0	3333.0	2411.9	3200.0

^a^ Medical comorbidity index on a scale of 0 (no medical comorbidities) to 15 (high comorbidities).

^b^ SMI defined as having a diagnosis of schizophrenia, schizoaffective disorder, bipolar disorder, or other psychoses.

^c^ Mortality proportion calculated as No. of deaths/No. of proportion^*^100,000.

^d^ Cancer mortality identified by ICD-10 codes for malignant neoplasm (C00–C97).

^e^ Cardiovascular-related mortality identified by ICD-10 codes for diseases of the heart (I00–I09, I11, I13, I20–I51).

^f^ Age- and sex standardized to the age and sex distribution of the non-Hispanic White FY2009 Veteran VHA user population.

AI/AN, American Indian/Alaskan Native; FY, fiscal year; NH/OPI, Native Hawaiian/Other Pacific Islander; SMI, serious mental illness; VHA, Veterans Health Administration.

### Mortality disparities within the VHA

#### Mortality proportions

Crude all-cause mortality proportions were highest for non-Hispanic Whites (11,486.9/100,000 persons), followed by NH/OPIs (9109.5/100,000 persons) (Table 1). Non-Hispanic Whites had the highest crude cancer and cardiovascular-related mortality.

All-cause mortality proportions (standardized to age/sex distribution of the non-Hispanic White FY2009 VHA users) were highest among AI/ANs (12,952.9/100,000 persons), followed by non-Hispanic Blacks (12,592.7/100,000 persons) (Table 1). Non-Hispanic Blacks had the highest age- and sex-standardized cancer and cardiovascular-related mortality.

#### Adjusted all-cause mortality

Table 2 presents adjusted Cox regression results. In age- and sex-adjusted models, all-cause mortality was higher for AI/ANs (HR=1.15, 95% CI: 1.10–1.21) and non-Hispanic Blacks (HR=1.06, 95% CI: 1.05–1.07) versus non-Hispanic Whites. After further medical and mental health comorbidity adjustment, the difference in all-cause mortality between AI/AN and non-Hispanic White Veterans persisted (HR=1.11, 95% CI: 1.06–1.16), whereas the difference for non-Hispanic Black was no longer statistically significant at the 0.05 level.

**Table 2.** Racial/Ethnic Differences in Veteran Health Administration User Adjusted Hazard Ratios for Mortality, by Mortality Type

	All-cause mortality		Cancer mortality		Cardiovascular-related mortality	
	Model 1	Model 2	Model 1	Model 2	Model 1	Model 2
Non-Hispanic White	Ref.	Ref.	Ref.	Ref.	Ref.	Ref.
AI/AN	**1.15** (1.10–1.21)	**1.11** (1.06–1.16)	**1.11** (1.02–1.21)	1.06 (0.98–1.16)	0.98 (0.89–1.08)	0.95 (0.86–1.04)
Asian	**0.61** (0.58–0.64)	**0.67** (0.64–0.70)	**0.66** (0.60–0.72)	**0.74** (0.68–0.81)	**0.56** (0.51–0.62)	**0.61** (0.56–0.67)
Non-Hispanic Black	**1.06** (1.05–1.07)	1.00 (0.99–1.01)	**1.15** (1.13–1.16)	**1.08** (1.06–1.10)	**1.10** (1.08–1.12)	**1.04** (1.02–1.05)
Hispanic	**0.84** (0.83–0.85)	**0.83** (0.82–0.84)	**0.76** (0.73–0.78)	**0.77** (0.74–0.79)	**0.75** (0.72–0.77)	**0.73** (0.71–0.76)
NH/OPI	0.99 (0.95–1.02)	**0.95** (0.91–0.99)	1.02 (0.95–1.10)	0.99 (0.92–1.07)	1.01 (0.94–1.09)	0.98 (0.91–1.05)

Bold denotes statistically significant difference from reference group (non-Hispanic White) at *p*<0.05.

Model 1 controlled for age and sex. Model 2 controlled for age, sex, and medical and mental health comorbidities.

Compared with non-Hispanic Whites, age- and sex-adjusted all-cause mortality was lower for Asians (HR=0.61, 95% CI: 0.58–0.64) and Hispanics (HR=0.84, 95% CI: 0.83–0.85). Differences remained after adjusting for medical and mental health comorbidity (Asians HR=0.67, 95% CI: 0.64–0.70; Hispanics HR=0.83, 95% CI: 0.82–0.84). NH/OPIs had lower age-, sex-, and comorbidity-adjusted all-cause mortality than non-Hispanic Whites (HR=0.95, 95% CI: 0.91–0.99).

#### Adjusted cancer mortality

After adjusting for age and sex, cancer mortality was higher among AI/ANs (HR=1.11, 95% CI: 1.02–1.21) and non-Hispanic Blacks (HR=1.15, 95% CI: 0.73–0.78) compared with non-Hispanic Whites. However, after adjusting for medical and mental health comorbidities, there was no statistically significant difference in cancer mortality between AI/ANs and non-Hispanic Whites. Cancer mortality was attenuated, although still statistically significantly higher among non-Hispanic Blacks than non-Hispanic Whites (HR=1.08, 95% CI: 1.06–1.10). Age-, sex-, and comorbidity-adjusted cancer mortality was lower among Asians (HR=0.74, 95% CI: 0.68–0.81) and Hispanics (HR=0.77, 95% CI: 0.74–0.79) versus non-Hispanic Whites.

#### Adjusted cardiovascular-related mortality

Cardiovascular-related mortality was higher in non-Hispanic Blacks than in non-Hispanic Whites after age and sex adjustment (HR=1.10, 95% CI: 1.08–1.12). Disparities persisted after comorbidity adjustment (HR=1.04, 95% CI: 1.02–1.05). After adjusting for age, sex, and comorbidities, Asians (HR=0.61, 95% CI: 0.55–0.67) and Hispanics (HR=0.72, 95% CI: 0.69–0.74) had lower cardiovascular-related mortality compared with non-Hispanic Whites.

### Comparison of VHA user mortality disparities with U.S. population disparities

**FIG. 1.**
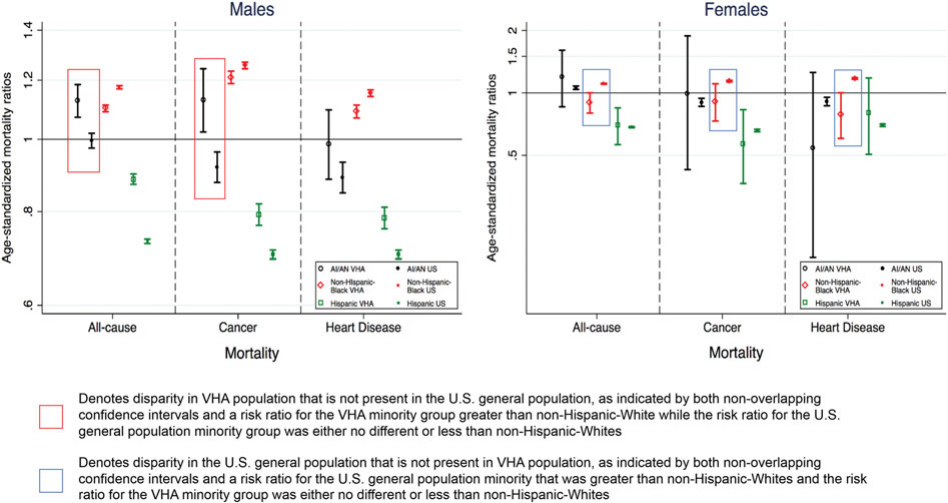
Comparison of age-standardized mortality risk ratios for the VHA and U.S. general populations by race/ethnicity compared with non-Hispanic Whites for males and females. Notes: point estimates and 95% CIs are presented for sex-stratified, age-standardized risk ratios for each racial/ethnic group compared with non-Hispanic Whites by population. Age standardized to the FY2009 non-Hispanic White VHA user population by sex. Asian and NH/OPI groups are not included as disaggregated mortality data from the U.S. general population are unavailable. AI/AN, American Indian/Alaskan Native; CI, confidence interval; NH/OPI, Native Hawaiian/Other Pacific Islander; FY, fiscal year; VHA, Veterans Health Administration.

Sex-stratified, age-standardized mortality proportions, used to calculate RR comparisons between the VHA and U.S. populations, and sex-stratified crude mortality ratios for the U.S. general population are included in Appendix Tables A1 and A2, respectively. Figure 1 presents sex-stratified, age-standardized RRs for the VHA cohort and U.S. general population. RR and confidence interval values are available in Appendix Table A3.

#### Age-standardized all-cause mortality

Within the U.S. general population, there was a non-Hispanic Black–non-Hispanic White disparity in mortality for women (RR=1.11; 95% CI: 1.10–1.11) that was not present for women in VHA (RR=0.90, 95% CI: 0.80–1.00). In contrast, there was an AI/AN-non-Hispanic White disparity in mortality for men in VHA (RR=1.13, 95% CI: 1.07–1.18) that was not present for the U.S. general population (RR=1.00, 95% CI: 0.97–1.02). There was a non-Hispanic Black–non-Hispanic White disparity in mortality for men in both U.S. and VHA populations; however, the disparity was somewhat lower in VHA.

#### Age-standardized cancer mortality

While a non-Hispanic Black–non-Hispanic White cancer mortality disparity existed for women in the U.S. general population (RR=1.14, 95% CI: 1.13–1.15), there was no statistically significant difference among non-Hispanic Black women VHA users (RR=0.91, 95% CI: 0.73–1.11). As with all-cause mortality, there was a disparity in cancer mortality for AI/AN men in the VHA (RR=1.13, 95% CI: 1.02–1.24) that was not present for the U.S. general population (RR=0.92, 95% CI: 0.88–0.96). Non-Hispanic Black–non-Hispanic White disparities among men were present in both the U.S. and VHA populations but were lower in the VHA.

#### Age-standardized cardiovascular-related mortality

While there was a non-Hispanic Black–non-Hispanic White disparity in cardiovascular-related mortality for women in the U.S. general population (RR=1.17, 95% CI: 1.16–1.18), there was no disparity between non-Hispanic Black and non-Hispanic White women within the VHA (0.79, 95% CI: 0.60–1.00). As with the other mortality causes, non-Hispanic Black–non-Hispanic White disparities among men were present in both the U.S. and VHA populations but were lower in the VHA.

## Discussion

We examined racial/ethnic mortality disparities within VHA, an equal-access health care system with additional characteristics to address nonfinancial access, and compared disparities within the VHA and U.S. general population. We found that within VHA, after adjusting for comorbidities, there were few racial/ethnic disparities in all-cause, cancer, and cardiovascular-related mortality. When compared with the U.S. general population, disparities were fewer and attenuated within VHA. However, disparities persisted within VHA in AI/AN all-cause mortality, and non-Hispanic Black cancer and cardiovascular-related mortality compared with non-Hispanic Whites. By contrast, all-cause and cause-specific mortality disparities among non-Hispanic Black women in the U.S. general population were not present in VHA.

Our finding that AI/ANs experienced all-cause mortality disparities compared with non-Hispanic Whites adds to limited research on VHAs AI/ANs disparities. More research to identify causes of AI/AN disparities within VHA is needed.^9^ Prior research has found that AI/ANs were more likely than other racial/ethnic groups to reside in highly rural areas,^11^ and had greater health care utilization barriers and unmet health care needs versus non-Hispanic White VHA users.^14^

Our finding of a mortality difference among AI/AN men relative to non-Hispanic Whites in the VHA but not in the U.S. general population should be interpreted cautiously. First, it is possible that VHA may see sicker AI/AN patients than those in the general population. Nearly one-fourth of Indian Health Service enrollees also use VHA, but primarily for specialty care.^15^ Second, under-reporting of AI/AN deaths^16^ may underestimate AI/AN disparities in both VHA and the U.S. general population, potentially to a larger degree in the U.S. general population due to a larger AI/AN sample size and proportion compared with VHA.

Among non-Hispanic Black VHA users, despite absence of a disparity in all-cause mortality, disparities existed in cancer and cardiovascular-related mortality relative to non-Hispanic Whites after comorbidity adjustment. Prior research found that non-Hispanic Black VHA users had similar or reduced nonsmall-cell lung and prostate cancer mortality—both important causes of cancer mortality in men—relative to non-Hispanic Whites.^17–19^ Mortality disparities in other types of cancers may explain discrepancy between our findings and existing studies. Our findings of cardiovascular-related mortality disparities are consistent with the existing evidence of higher cardiovascular disease risk factors and worse control versus non-Hispanic Whites.^11,20,21^ While VHAs behavioral risk factor reduction programs (e.g., smoking cessation and weight management) could potentially alter both cardiovascular and cancer risks, these programs are underutilized.^22,23^

Despite VHA efforts to improve care for all eligible Veterans, more research is needed to understand additional contributors to AI/AN and non-Hispanic Black mortality disparities. These may include differences in health behaviors (e.g., tobacco use), culturally appropriate care, use of lower performing VHA facilities, social determinants of health (e.g., socioeconomic status and neighborhood resources), and structural racism and discrimination. Structural racism, which operates through social forces and political institutions, includes residential, school, and workplace segregation, historic trauma (e.g., slavery), and policing and incarceration of racial/ethnic minorities.^24–27^ Discrimination may occur in medical settings, resulting in provider biases that affect how they interact with and treat racial/ethnic minorities,^28^ or nonmedical settings, resulting in allostatic load, metabolic disruption, and worse mental health.^29^ VHA is working to address some—though not all—of these factors, such as job training, housing support, and through VA Office of Health Equity, cultural competency.^11,30,31^

Our finding of mortality differences among AI/ANs and non-Hispanic Blacks versus non-Hispanic Whites *before* comorbidity adjustment, which were no longer statistically significant after comorbidity adjustment, suggests that these groups may have similar mortality rates within a similar level of health. However, AI/ANs and non-Hispanic Blacks may have more underlying comorbidities that negatively affect their health-related quality of life (i.e., mortality differences may reflect confounding by comorbidity). Closing gaps in underlying comorbidities may require targeted primary and secondary prevention efforts, and ensuring high-quality care in VHA facilities predominately serving these groups.

One of the challenges of studying NH/OPI mortality disparities in the U.S. general population is that national datasets often combine NH/OPIs with Asians. VHA's data uniquely allowed us to examine disparities in this understudied group, but we could not compare disparities with the U.S. general population: CDC mortality files do not disaggregate NH/OPIs from Asians. Our finding that NH/OPIs in VHA had comparable cancer and cardiovascular-related mortality, and lower all-cause mortality relative to non-Hispanic Whites adds to existing limited—and inconsistent—findings about NH/OPIs health disparities in VHA.^21^

We found that disparity patterns differed between non-Hispanic Black men and women. Non-Hispanic Black men in both the U.S. general population and VHA experienced greater mortality than non-Hispanic White men, but this difference was smaller in VHA. This suggests that VHA efforts to improve access may help close some of the disparities, but other contributors still should be explored. In contrast, among non-Hispanic Black women, we found disparities in the U.S. general population but not in VHA. Women comprise a small, but growing group of patients within VHA. VHA has made recent strides in adapting programs to women Veterans.^32^ More research is needed to understand how characteristics of men and women VHA users (e.g., age, mental illness prevalence, reasons for joining military) influence disparate racial/ethnic disparities, and whether aspects of VHA care contribute to mitigating non-Hispanic Black–non-Hispanic White mortality disparities in women.

This work had several limitations. Race/ethnicity misclassification may have occurred in the CDC mortality files, particularly for AI/ANs.^16^ CDC mortality files do not disaggregate NH/OPIs from Asians. The comparison of mortality ratios between the VHA and U.S. general population cohorts may be sensitive to the population to which we chose to standardize our analyses. Although our findings of distinct racial/ethnic mortality disparities in VHA and the U.S. general population may reflect some impact of VHA health care, residual confounding driven by differences between the VHA and the U.S. general populations (e.g., comorbidity, health status, age),^33^ or self-selection bias in who chooses to serve in the military and/or use VHA care may be important. For example, Veteran VHA users have lower incomes and greater service-connected disability than Veteran non-VHA-users,^34,35^ which could potentially increase VHA–U.S. disparities, but reduce within-VHA disparities. Finally, we could not control for comorbidity severity (e.g., cancer stage).

## Conclusion

VHA's success in delivering care to racial/ethnic groups that experience mortality disparities in the U.S. general population highlights the value of its efforts, as an equal-access system that strives to make comprehensive, integrated care available to all members, in addressing persistent racial/ethnic disparities. Despite differences between the U.S. and VHA populations (e.g., VHA users had prior military exposure, more chronic^36^ and mental health conditions,^37^ and within this study, at least 1 health care visit), implications of this analysis extend beyond VHA to inform how equal-access systems, and broader efforts to expand access to integrated, comprehensive care, may mitigate racial/ethnic health disparities. Other health systems—even nonequal-access systems—can aim to emulate VHA's efforts to make health care accessible. Broader policy initiatives to increase health care access and reduce financial barriers, such as insurance expansion, may also help eliminate disparities.

More work is needed to understand and address residual disparities within VHA, particularly in all-cause mortality among AI/ANs, and cancer and cardiovascular-related mortality among non-Hispanic Blacks, including improving quality of care and appropriate utilization within VHA, and examining nonhealth care factors, including health behaviors and social determinants of health (e.g., socioeconomic status) in explaining residual racial/ethnic disparities in mortality within VHA.

## Supplementary Material

Supplemental data

## Abbreviations Used

AI/ANs: American Indian/Alaskan Natives
CDC: Centers for Disease Control and Prevention
CI: confidence interval
FY: fiscal year
HR: heart rate
NCHS: National Center for Health Statistics
NDI: National Death Index
NH/OPIs: Native Hawaiians/Other Pacific Islanders
RR: risk ratios
SMI: serious mental illness
VHA: Veterans Health Administration

## Appendix

**Appendix Table A1.** Sex-Stratified, Age-Standardized Mortality Proportions for the Veterans Health Administration and U.S. Adult General Population

	All-cause mortality		Cancer mortality		Cardiovascular-related mortality	
	VHA cohort	U.S. general population	VHA cohort	U.S. general population	VHA cohort	U.S. general population
Men						
Non-Hispanic White	0.118	0.034	0.030	0.008	0.030	0.009
AI/AN	0.133	0.040	0.034	0.008	0.029	0.008
Non-Hispanic Black	0.130	0.025	0.037	0.010	0.030	0.011
Hispanic	0.104	0.025	0.024	0.006	0.023	0.007
Women						
Non-Hispanic White	0.048	0.014	0.011	0.003	0.010	0.004
AI/AN	0.058	0.015	0.011	0.003	0.005	0.003
Non-Hispanic Black	0.043	0.016	0.010	0.003	0.008	0.004
Hispanic	0.034	0.010	0.006	0.002	0.006	0.002

Authors' analysis of CDC NDI data as provided by the VA/DoD Suicide Data Repository, and mortality files from the NCHS, with age-adjusted using U.S. Census annual population estimates.

Values represent mortality proportions (i.e., mortality count/population count), using age standardization weights based on the FY2009 non-Hispanic White VHA user population by sex.

**Appendix Table A2.** Sex-Stratified Crude Mortality Ratios per 100,000 Persons for the U.S. Adult General Population

	All-cause mortality		Cancer mortality		Cardiovascular-related mortality	
	Men	Women	Men	Women	Men	Women
Non-Hispanic White	1265.8	443.7	314.9	269.1	323.8	290.3
AI/AN	982.8	795.4	184.1	153.8	197.0	138.5
Non-Hispanic Black	1076.0	919.8	259.8	215.6	267.5	224.5
Hispanic	443.7	374.1	96.5	87.7	95.3	81.1

Authors' analysis of mortality files from the NCHS, with age-adjusted using U.S. Census annual population estimates.

**Appendix Table A3.** Comparison of Sex-Stratified, Age-Standardized Mortality Risk Ratios for the Veterans Health Administration and U.S. General Populations

	All-cause mortality		Cancer mortality		Cardiovascular-related mortality	
	VHA cohort	U.S. general population	VHA cohort	U.S. general population	VHA cohort	U.S. general population
Men						
Non-Hispanic White	Ref.	Ref.	Ref.	Ref.	Ref.	Ref.
AI/AN	***1.13****(1.07–1.18)*	*1.00 (0.97–1.02)*	***1.13****(1.02–1.24)*	***0.92****(0.88–0.96)*	0.99 (0.88–1.09)	**0.89** (0.85–0.93)
Non-Hispanic Black	***1.10****(1.09–1.11)*	***1.17****(1.17–1.18)*	***1.21****(1.19–1.23)*	***1.26****(1.24–1.27)*	***1.09****(1.07–1.11)*	***1.15****(1.14–1.16)*
Hispanic	***0.88****(0.87–0.90)*	***0.73****(0.73–0.73)*	***0.79****(0.77–0.82)*	***0.70****(0.69–0.71)*	***0.78****(0.76–0.81)*	***0.70****(0.69–0.71)*
Women						
Non-Hispanic White	Ref.	Ref.	Ref.	Ref.	Ref.	Ref.
AI/AN	1.19 (0.86–1.61)	**1.06** (1.04–1.08)	0.99 (0.43–1.88)	**0.90** (0.86–0.94)	0.54 (0.16–1.26)	**0.91** (0.87–0.95)
Non-Hispanic Black	*0.90 (0.80–1.0001)*	***1.11****(1.10–1.11)*	*0.91 (0.73–1.11)*	***1.14****(1.13–1.15)*	*0.79 (0.60–1.001)*	***1.17****(1.16–1.18)*
Hispanic	**0.70** (0.56–0.85)	**0.68** (0.68–0.69)	**0.57** (0.37–0.83)	**0.66** (0.65–0.67)	0.80 (0.51–1.18)	**0.70** (0.69–0.71)

Authors' analysis of CDC NDI data as provided by the VA/DoD Suicide Data Repository, and mortality files from the NCHS, with age-adjusted using U.S. Census annual population estimates.

Within a column, a test of the significance of a minority/non-Hispanic White disparity in mortality is presented, where a standardized RR in bold indicates a statistically significant difference from the reference group (non-Hispanic White) within each population at *p*<0.05.

Within a row, a test of the significance of the difference in the magnitudes of a standardized RR for the VHA cohort and a standardized RR for the U.S. general population are presented, where the RRs in italics denote nonoverlapping CIs between the VHA cohort and U.S. general population.

Age standardized to the FY2009 non-Hispanic White VHA user population by sex.

AI/AN, American Indian/Alaskan Native; CDC, Centers for Disease Control and Prevention; CI, confidence interval; FY, fiscal year; NCHS, National Center for Health Statistics; NDI, National Death Index; RR, risk ratio; VHA, Veterans Health Administration.
