# Supplementary material for: Racial/Ethnic Disparities in Mortality Across the Veterans Health Administration
Source: Health Equity. 2019 Apr 8;3(1):99–108. doi: 10.1089/heq.2018.0086 (PMC6608703; doi:10.1089/heq.2018.0086)
Supplement: Supplemental data [file Supp_Data.pdf]

Supplementary Data

Details of Analytical Sample

Our full cohort of 5,140,379 Veterans was reduced to an analytical sample size of 5,030,722 in the following way: first, we excluded data from 13 Veterans on whom National Death Index had duplicated mortality records, thus reducing the sample to 5,140,366. In addition, time to mortality could not be computed on 3866 Veterans because their dates of death preceded their ambulatory care visit dates, thus reducing the sample size to 5,136,500. Furthermore, data from an additional 104,879 Veterans were excluded, because these Veterans had no ambulatory care visits in the 2009 fiscal year, thus resulting in missing time-to-mortality measurements. This reduced the sample size to 5,031,621. There were 899 Veterans whose dates of death were the same as their ambulatory care visit dates, which reduced the sample size to 5,030,722. Finally, 66 Veterans were excluded because of missing covariates, thus reducing the final analytical sample size to 5,030,656.
